# Endocytosis: a pivotal pathway for regulating metastasis

**DOI:** 10.1038/s41416-020-01179-8

**Published:** 2020-12-02

**Authors:** Imran Khan, Patricia S. Steeg

**Affiliations:** Women’s Malignancies Branch, Center for Cancer Research, National Cancer Institute, Bethesda, MD 20892 USA

**Keywords:** Metastasis, Breast cancer, Cell invasion, Bone metastases

## Abstract

A potentially important aspect in the regulation of tumour metastasis is endocytosis. This process consists of internalisation of cell-surface receptors via pinocytosis, phagocytosis or receptor-mediated endocytosis, the latter of which includes clathrin-, caveolae- and non-clathrin or caveolae-mediated mechanisms. Endocytosis then progresses through several intracellular compartments for sorting and routing of cargo, ending in lysosomal degradation, recycling back to the cell surface or secretion. Multiple endocytic proteins are dysregulated in cancer and regulate tumour metastasis, particularly migration and invasion. Importantly, four metastasis suppressor genes function in part by regulating endocytosis, namely, the NME, KAI, MTSS1 and KISS1 pathways. Data on metastasis suppressors identify a new point of dysregulation operative in tumour metastasis, alterations in signalling through endocytosis. This review will focus on the multicomponent process of endocytosis affecting different steps of metastasis and how metastatic-suppressor genes use endocytosis to suppress metastasis.

## Background

Cancer is the second leading cause of global mortality.^1^ The spread of cancer cells from the primary tumour to distant organs and their subsequent progressive colonisation is referred to as metastasis. It is estimated that 90% of cancer-related deaths are due to metastatic disease rather than to the primary tumour growth. Typically, treatments for metastatic cancer are systemic therapy involving chemotherapy or molecular drugs, hormonal agents, immune checkpoint drugs, radiation therapy or surgery. Despite progress in extending cancer-survivorship rates,^2^ limited progress has been made in the treatment of metastatic cancer due to its complex nature and an inadequate understanding of the molecular and biochemical mechanisms involved.

Metastasis is a multistep process involving tumour cell invasion to neighbouring areas, intravasation into the bloodstream, arrest in the capillary bed of a secondary organ, extravasation from the circulatory system and colonisation at the secondary site.^3^ All of the above steps occur via complex interactions between cancer cells and their microenvironments. Despite the documented complexity and redundancy of the metastatic process, mutation or changes in the expression of single genes have been reported to alter metastatic ability. Genes that are involved in the promotion of metastasis at distant sites are referred to as metastasis promoting genes. Expression of these genes facilitates cancer cell establishment of appropriate interactions with changing microenvironments to promote continued survival and proliferation at secondary sites. Similarly, genes that inhibit the process of metastasis without affecting the growth of the primary tumour are referred to as metastasis suppressor genes and are described in detail in the later part of this review.

This review will highlight an often-overlooked aspect of metastasis, receptor endocytic pathways. Contributing to each step in metastasis is the distribution of multiple cell-surface receptors on tumour and microenvironmental cells. Receptor signalling is, in turn, modulated by endocytosis (internalisation, recycling or degradation). In recent years, there has been significant progress made towards understanding the mechanisms of the endocytosis pathway and its alterations that occur during metastasis. A growing body of literature suggests that receptor endocytosis affects metastasis and could be a tool for the functioning of metastasis suppressor or metastasis promoters. This review will focus on the role of endocytosis in metastasis and how these pathways are used by metastasis suppressors.

## Endocytic pathways and metastasis

The term ‘endocytosis’ is derived from the Greek word ‘endon’, meaning within, ‘kytos’, meaning cell and ‘-osis’, meaning process. So, endocytosis is the process by which cells actively internalise molecules and surface proteins via an endocytic vesicle. Depending on the cargo type, internalisation route and scission mechanism, there are three general modes of vesicular endocytic trafficking that coexist in the cell and operate concurrently: phagocytosis, pinocytosis and receptor-mediated endocytosis. In phagocytosis, the cell’s plasma membrane surrounds a macromolecule (large solid particles > 0.5 μm) or even an entire cell from the extracellular environment and generates intracellular vesicles called phagosomes.^4^ Cellular pinocytosis/cellular drinking is a process in which fluids and nutrients are ingested by the cell, by pinching in and forming vesicles that are smaller than the phagosomes (0.5–5 μm).^5^ Both phagocytosis and pinocytosis are non-selective modes of taking up molecules. However, there are times when specific molecules are required by cells and are taken up more efficiently by the process of receptor-mediated endocytosis (RME). The endocytosis of specific cargoes via specific receptors can take place by clathrin-mediated (CME), caveolae-mediated (CavME), clathrin- and caveolae-independent endocytic (CLIC/GEEC) pathways. These endocytic pathways are briefly described below. Table 1 links selected endocytic proteins to in vitro components of the metastatic process and in vivo metastasis in cancer.

**Table 1:** Validated roles of endocytic proteins in metastasis.

Endocytic protein	Function(s)	Phenotypic effects (in vitro, unless noted)	Cancer types studied
Clathrin-mediated endocytosis (CME)			
AP2	Recruits cargo and clathrin to growing clathrin coated pits	Modulates tumour cell migration, invasion and chemotaxis through CXCR-2.	Ovarian, pancreatic and melanoma cancer^89^
Clathrin	Component of the coat protein for membrane invagination in endocytosis	Clathrin light chain isoform (CLCb) is upregulated and associated with poor prognosis. NSCLC cells expressing CLCb exhibit increased cell migration and the metastasis (in vivo).	NSCLC^17^
Dynamin	Dynamins are large GTPase, encoded by three genes in mammals, required for scission of newly formed vesicles from the membrane	Dynamin 1 and 2 are known to enhance cancer cell proliferation, tumour invasion and metastasis, whereas Dynamin 3 has a tumour-suppressive role.	NSCLC cells^90^
		Dynamin 2 overexpression correlates with poor prognosis.	Prostate cancer^18^
Caveolin-mediated endocytosis (CavME)			
Caveolin	Major coat protein of caveolae and involved in invagination of lipid raft domain	In early stages of the disease caveolin functions predominantly as a tumour suppressor, whereas at later stages its expression is associated with tumour progression and metastasis. The late-stage tumour progression and metastasis effect of CAV-1 has been attributed to tyrosine (Tyr14) phosphorylation of its protein product by Src kinases.	Hepatomas, ovarian cancers, prostate cancer and breast cancer^30,31^
		CAV-1 is often deleted in human cancers and acts by inhibiting cytokine receptor signalling.	Breast cancer^31,32^
		Knockdown of CAV-1 reduced velocity, directionality and persistency of cellular migration.	Breast and prostate cancers^31,32^
		Positive expression of CAV-1 is a marker of histopathological grade and poor prognosis (pancreatic cancer). Low expression of CAV-1 is associated with poor prognosis (hepatic cancer).	Pancreatic adenocarcinoma and lung cancer; hepatocellular carcinoma^33^
Clathrin-independent endocytosis (CIE)	The endocytic vesicles involved in CIE have no distinct coat	CIE pathway suppresses cancer cell blebbing and invasion through GTPase-activating protein GRAF1.	Colon cancer^39^
Endosomal trafficking proteins			
ARF subfamily:	Small GTPase family		
ARF1	Regulates assembly of different types of ‘coat’ complexes onto budding vesicles, involved in secretory pathway and activates lipid-modifying enzymes	Controls cellular migration and proliferation by regulating interaction between β1-integrin and key proteins of focal adhesions, such as paxillin, talin and FAK.	Breast cancer^91,92^
ARF4	Regulates retrograde transport from endosomes to the TGN	Tensin-mediated cellular invasion and migration is modulated by ARF4-dependent internalisation of α5β1-integrins to late endosomes/lysosomes and consequently their degradation.	Ovarian cancer^93^
ARF6	Regulates endocytic membrane trafficking and actin remodelling	ARF6 promotes E-cadherin internalisation and facilitates disassembly of adherens junction for cellular motility and invasion.	MDCK cells,^94,95^ glioma, breast cancer^96^
		ARF6 inhibitor impairs melanoma pulmonary metastasis.	Melanoma^97^
Ras-homologue (RHO) subfamily:			
RHOA	Facilitates the assembly of contractile actomyosin filaments in focal adhesion complexes and vesicle trafficking	Loss of RHOA expression prevents the endocytosis of multiple receptors and enhances breast cancer metastasis in vivo with a concomitant increase in CCR5 and CXCR4 chemokine signalling.	Breast cancer^98^
		N-WASP regulates endosomal recycling of LPAR1 which increases RhoA-mediated contractile responses, cell steering and spontaneous metastasis. Mice with N-WASP-depleted tumours survived significantly longer.	Pancreatic ductal adenocarcinoma^99^
RAC1	Regulates macropinocytosis, membrane trafficking and cellular morphology	RAC1 activation leads to cancer cell proliferation/survival, actin remodelling/migration (EMT transition phenotype), metastasis in vivo and angiogenesis.	Breast cancer,^100,101^ gastric adenocarcinoma^102^
CDC42	Involved in intracellular trafficking, ER–Golgi interface trafficking (both anterograde and retrograde), post-Golgi transport and exocytosis	Activation/overexpression promoted tumour progression and metastasis in different tumour types in vivo.	NSCLC, gastric cancer, breast cancer^103^
RAB subfamily:			
RAB1 (RAB1A and RAB1B)	Regulates ER-Golgi traffic	Loss of RAB1B expression in triple-negative breast cancer correlates with higher metastasis.	Breast cancer,^104^ NSCLC, gastric cancer, and oesophageal squamous cell carcinoma^105^
RAB2	Retrograde transfer of vesicles	RAB2A overexpression causes increased cellular invasiveness and acquisition of EMT traits.	Breast cancer^106^
RAB3	Modulates secretion of vesicles leading to exocytosis	RAB3C overexpression promotes migration, invasion and metastasis in vivo.	Colorectal cancer^107^
		RAB3D promotes breast cancer cell invasion and lung metastasis in vivo through activation of the AKT/GSK-3β/Snail signalling pathway.	Breast cancer^108^
RAB4	Regulates recycling of vesicles	RAB4 recycling route is central in promoting invasive properties of cancer cells through integrin β3.	Breast cancer^109^
RAB5, RAB21 and RAB22	Regulates early endosome trafficking	RAB5 promotes integrin trafficking, focal adhesion turnover, RAC1 activation, tumour cell migration and invasion.	Breast cancer,^110^ colon adenocarcinoma and melanoma^111^
		RAB5 mediates hypoxia-driven tumour cell migration, invasion and metastasis in vivo.	Breast cancer and melanoma^112^
		RAB21 regulates integrin-mediated cell adhesion and motility.	Cervical cancer cells^113^
		RAB22 and RAB163 (C-terminal of BRCA2 protein) specifically interact with the RAD51 protein.	Affects breast cancer susceptibility gene (BRCA2)^114^
		TBC1D2b (a RAB22 GTPase-activating protein) is suppressed by ZEB1/NuRD complex to increase E-cadherin internalisation and promote metastasis in lung cancer using subcutaneous syngeneic mice model.	NSCLC^115^
RAB6	Regulates anterograde and retrograde trafficking routes between the Golgi apparatus, endoplasmic reticulum, plasma membrane, and endosomes	Elevated expression of RAB5A correlates with either poor or favourable prognosis.	Colorectal cancer,^116^ gastric cancer^117^
		Knockdown promotes cell migration by inhibiting myosin II phosphorylation and upregulating Cdc42 activity.	Bone osteosarcoma, NSCLC cells^118^
RAB7	Regulates late endocytic pathway, including endosome maturation, early endosomes to late endosomes transition, clustering and fusion to lysosomes	RAB7 downregulation is important for acquisition of invasive properties in melanoma cells and correlates with increased risk of metastasis development.	Melanoma^119^
RAB11	Regulates membrane protein recycling and protein transport from TGN to the plasma membrane (slow recycling)	Regulates RAC activity and polarisation during collective cell migration, hypoxia-stimulated cell invasion in cancer cells.	Breast cancer^120^
		RCP (a RAB11 effector)-dependent trafficking of Eph receptor drives cell–cell repulsion and metastasis in an autochthonous mouse model of pancreatic adenocarcinoma.	Pancreatic adenocarcinoma^121^
RAB27	Regulates secretory pathway/exocytosis and melanosomes	Both the isoforms RAB27A and RAB27B, are known to promote cell invasion and tumour metastasis in vivo.	Bladder cancer, melanoma and breast cancer cells^122^
RAB35	Regulates fast recycling of proteins to the plasma membrane and in sorting endosomes	Constitutively active RAB35 is proposed to be oncogenic due to activation of PI3K/Akt signalling. Regulates cancer cell migration and invasion.	Gastric cancer, cervical cancer cells^123^
		Mutant p53 drives metastasis in autochthonous mouse models of pancreatic cancer by controlling the production of sialomucin, podocalyxin and activity of the RAB35 GTPase, which interacts with podocalyxin to influence its sorting to exosomes. These exosomes influence integrin trafficking in normal fibroblasts to promote deposition of a highly pro-invasive ECM.	Pancreatic cancer and NSCLC^124^

*ECM* extracellular matrix, *PI3K* phosphoinositide 3-kinase, *RCP* RAB-coupling protein, *TGN* trans-Golgi network, *GSK-3β* glycogen synthase kinase 3β, *EMT* epithelial-to-mesenchymal transition, *CXCR-2* C-X-C chemokine receptor-2, *NSCLC* non-small cell lung cancer, *FAK* focal adhesion kinase, *CCR5* CC- chemokine receptor 5.

### Clathrin-mediated endocytosis (CME)

The most studied endocytic mechanism is CME. It was first found to play an important role in low-density lipoprotein^6^ and transferrin uptake.^7^ It is known to be involved in internalisation and recycling of multiple receptors engaged in signal transduction (G-protein and tyrosine-kinase receptors), nutrient uptake and synaptic vesicle reformation.^8^ Clathrin-coated pits (CCP) are assemblies of cytosolic coat proteins, which are initiated by AP2 (assembly polypeptide 2) complexes that are recruited to a plasma membrane region enriched in phosphatidylinositol-(4,5)-bisphosphate lipid.^9^ AP2 acts as a principal cargo-recognition molecule and recognises internalised receptors through a short sequence motif in their cytoplasmic domains.^10^ As the nascent invagination grows, AP2 and other cargo-specific adaptor proteins recruit and concentrate the cargo, which is now facing the inside of the vesicle. Following cargo recognition/concentration, AP2 complexes along with other adaptor proteins to recruit clathrin. Clathrin recruitment stabilises the curvature of the growing CCP with the help of BAR (Bin-Amphiphysin-Rvs)-domain-containing proteins until the entire region invaginates to form a closed vesicle.^11^

Release of mature clathrin-coated vesicles from the plasma membrane is performed by the large multi-domain GTPase, Dynamin. Proteins such as amphiphysin, endophilin and sorting nexin 9 (BAR-domain-containing proteins) recruit Dynamin around the necks of budding vesicles.^12^ Similarly, other Dynamin partners (i.e., Grb2) also bind to Dynamin and increase its oligomerisation, which results in a higher GTPase activity.^13^ Oligomerised Dynamin assembles into collar-like structures encircling the necks of deeply invaginated pits and undergoes GTP hydrolysis to drive membrane fission.^14^ After a vesicle is detached from the plasma membrane, the clathrin coat is disassembled by the combined action of the ATPase HSC70 and the coat component auxilin.^15,16^ The released uncoated vesicle is ready to travel and fuse with its target endosome.

Signalling through CME is critical in cancer and metastasis. Clathrin light-chain isoform (CLCb) is specifically upregulated in non-small-cell lung cancer (NSCLC) cells and is associated with poor prognosis. NSCLC cells expressing CLCb exhibit increased rates of CME through Dynamin 1. This leads to activation of a positive feedback loop involving enhanced epidermal growth factor receptor (EGFR)-dependent Akt/GSK-3β (glycogen synthase kinase 3β) phosphorylation, resulting in increased cell migration and metastasis.^17^ Dynamin 2 is crucial for the endocytosis of several proteins known to be involved in cancer motility and invasiveness (e.g., β-1 integrin and focal adhesion kinase). Dynamin 2 overexpression correlates with poor prognosis.^18^

The regulation of certain receptors that are known to affect cancer and metastasis (i.e., EGFR and transforming growth factor β receptor (TGFβR)) by clathrin- and non-clathrin-mediated internalisation pathways preferentially targets the receptors to different fates (i.e., recycling or degradation).^19,20^ Different fates of receptors determine the net signalling output in a cell and affect cancer progression. Interestingly, CME is known to skew EGFR fate towards recycling rather than degradation, leading to prolonged duration of signalling.^20^ Similarly, the internalised EGF–EGFR complex may maintain its ability to generate cell signalling from endosomes affecting multiple downstream pathways.^21^ This active endosomal EGFR is known to regulate oncogenic Ras activity by co-internalising its regulators including Grb2, SHC, GAP and Cbl.^21,22^

### Caveolae-mediated endocytosis (CavME)

CavME is the second most studied pathway of endocytosis and has been shown to be important in transcytotic trafficking across cells and mechanosensing.^23^ The CavME process involves formation of a bulb-shaped, 50–60-nm plasma membrane invaginations called caveolae (little caves), which is driven by both integral membrane proteins called caveolins and peripheral membrane proteins called cavins (cytosolic coat proteins). Caveolins (encoded by CAV-1, 2 and 3 paralogues) are small integral membrane proteins that are inserted into the inner side of the membrane bilayer through its cytosolic N-terminal region that binds to cholesterol. About 50 cavin molecules associate with each caveolae and exist in a homo- or hetero-oligomeric form (using four cavin family members).^24^ CavME is triggered by ligand binding to cargo receptors concentrated in caveolae. Budding of caveolae from the plasma membrane is regulated by kinases and phosphatases, such as Src tyrosine kinases and serine/threonine protein phosphatases PP1 and PP2A.^25^ As with CME, Dynamin is required to pinch off caveolae vesicles from the plasma membrane.^26^

Components of CavME have a vital role in cell migration, invasion and metastasis. It is speculated that CAV-1 has a dual role in cancer progression and metastasis. In the early stages of the disease, it functions predominantly as a tumour suppressor, whereas at later stages, its expression is associated with tumour progression and metastasis.^27–29^ As with a tumour suppressor, CAV-1 is often deleted in human cancers and mechanistically known to act through the caveolin scaffolding domain (CSD) by inhibiting cytokine receptor signalling.^28,30^ The CAV-1 effect on the late-stage tumour progression and metastasis has been attributed to tyrosine (Tyr14) phosphorylation of its protein product by Src kinases, leading to increased Rho/ROCK signalling and subsequent focal adhesion turnover.^31^ Knockdown of CAV-1 in breast and prostate cancer cells reduced the velocity, directionality and persistency of cellular migration.^31,32^ Similarly, expression of CAV-1 has been used as a marker of prognosis and overall survival in various types of human cancer. In pancreatic adenocarcinoma, positive expression of CAV-1 was found to correlate with tumour diameter, histopathological grade and poor prognosis. In lung cancer, CAV-1 expression statistically correlates with poor differentiation, pathological stage, lymph-node metastasis and poor prognosis. However, in hepatocellular carcinoma tissues, low expression of CAV-1 is associated with poor prognosis.^33^

### Clathrin-independent endocytosis (CIE)

As per the name, the endocytic vesicles involved in CIE have no distinct coat and were first discovered by their resistance to inhibitors that block CME and CavME.^34^ CIE encompasses several pathways. (i) An endophilin-, Dynamin- and RhoA-dependent pathway for endocytosis of interleukin-2 receptor.^35^ (ii) A clathrin- and Dynamin-independent (CLIC/GEEC) pathway in which the GTPases RAC1 and CDC42 lead to actin-dependent formation of clathrin-independent carriers (CLICs). This, in turn, forms the glycosylphosphatidylinositol (GPI)-AP-enriched endosomal compartments (GEECs).^36,37^ (iii) An ARF6-dependent pathway involving the small GTPase ARF6, to activate phosphatidylinositol-4-phosphate 5-kinase that produces phosphatidylinositol-(4,5)-bisphosphate, leading to stimulation of actin assembly and endocytosis.^38^ The CIE pathway has been shown to suppress cancer cell blebbing and invasion through GTPase-activating protein GRAF1 (GTPase regulator associated with focal adhesion kinase-1) (Table 1).^39^ Various receptors are endocytosed by the CIE pathway, including interleukin-2 receptor (IL-2R), T-cell receptor (TCR) and GPI-linked proteins.^40^

## Downstream endosomal trafficking

**Fig. 1:**
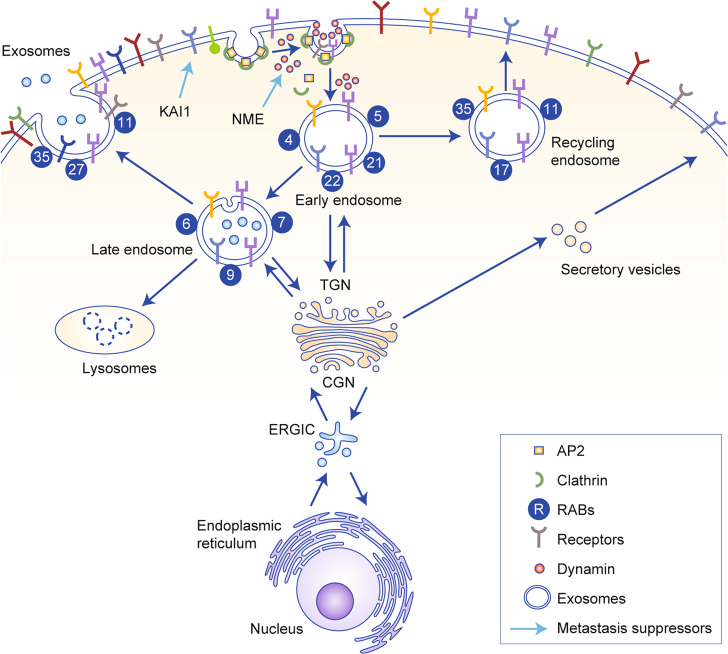
Endosomal trafficking and metastasis suppressor genes. A wide variety of receptors and their ligands are moved intracellularly by endocytosis. Clathrin-mediated endocytosis begins with initiation and maturation of clathrin-coated pits by AP2 complexes that are recruited to the plasma membrane and act as a principal cargo-recognition molecule. As the nascent invagination grows, AP2 and other cargo-specific adaptor proteins recruit and concentrate the cargo. AP2 complexes along with other adaptor proteins to recruit clathrin. Clathrin recruitment stabilises the curvature of the growing pit with the help of other BAR-domain-containing proteins. BAR-domain-containing proteins also recruit Dynamin to the neck of the budding vesicle, until the entire region invaginates to form a closed vesicle. Dynamin is a large GTPase, which forms a helical oligomer around the constricted neck and, upon GTP hydrolysis, mediates the fission of the vesicle to release it into the cytoplasm. Following vesicle detachment from the plasma membrane, the clathrin coat is disassembled. The released vesicle goes through a first set of fusion, leading to formation of early endosomes, where initial sorting decisions are made, and the fate of the internalised sorting proteins and lipids is decided. The RAB proteins primarily localised to the early endosome include RAB5 and RAB4, along with lesser-known RAB21 and RAB22. They regulate the motility of early endosome on actin and microtubule tracks, its homotypic fusion and specialised functions of sorting and trafficking. The internalised receptors can be sorted into recycling pathways through extensive tubulation of the early endosome membranes, wherein receptors that are sorted into the newly formed tubular membranes recycle back to the plasma membrane through recycling endosomes. Alternately, early endosome growth and maturation could lead to the *trans*-Golgi network (TGN) or to late endosomes. Mature late endosomes are approximately 250–1000 nm in diameter and are characterised by the generation of a RAB7 domain. Late endosomes undergo homotypic fusion reactions, grow in size and acquire more intralumenal vesicles (ILVs). ILVs containing late endosomes get enriched with RAB35 and RAB27 and their effectors that promote their fusion to plasma membrane to release exosomes (40–100 nm in diameter vesicles). Predominantly, late endosomes move to the perinuclear area of the cell where they undergo transient fusions with each other and eventually fuse with lysosomes for degradation of its content. Cellular proteins synthesised in the rough endoplasmic reticulum (ER) are constantly secreted from ER to the Golgi complex in mammals through an ER–Golgi intermediate compartment (ERGIC). Points where metastasis suppressors interact with the endocytic process are highlighted.

Internalised receptor–ligand cargoes can merge into a common endosomal network by undergoing multiple rounds of fusions. The first set of fusion leads to the formation of early endosomes where initial sorting routes are engaged, and the fate of the internalised receptors is decided (Fig. 1). Early endosomes are identified by the association of several proteins on their cytosolic surface, including RAB5, along with its effector VPS34/p150, a phosphatidylinositol 3-kinase complex. VPS34/p150 generates phosphatidylinositol 3-phosphate, which regulates the spatiotemporal and compartmentalisation aspects of endosomal functions.^41,42^ Structurally, early endosomes have tubular (membrane) and vacuolar (vacuoles) domains. Most of the membrane surface area lies in the tubules, while much of the volume is in the vacuoles. The membrane domains are enriched in proteins, including RAB5, RAB4, RAB11, ARF1/COPI, retromer and caveolin.^43,44^ These proteins are involved in multiple functions, including molecular sorting of early endosomes to distinct organelles, its recycling and maturation to late endosomes or to the *trans*-Golgi network (TGN) (Fig. 1). The role of these endocytic proteins in metastasis in vivo and their prognostic potential, if any, have been listed in Table 1.

A recycling pathway returns endosomes to the cell surface either by a fast recycling route (via RAB4-positive endosomes) or by a slow recycling route (via RAB11-positive endosomes).^45^ Internalised receptors in early endosomes can be sorted into the recycling pathway through an extensive tubulation of the early endosome membranes in a process called ‘geometry-based sorting’ wherein receptors that are sorted into the newly formed tubular membranes of the early endosome are recycled back to the plasma membrane. Intralumenal vesicles (ILVs) also form in early endosomes, driven by clathrin and components of the endosomal sorting complex required for transport (ESCRT).^46^ ESCRT-mediated receptor sorting into ILVs is an evolutionarily conserved process that is required for multivesicular body (MVB) formation. ESCRT uses its various complexes for receptor recognition (ESCRT-0), inward budding (ESCRT-I and II) and final ESCRT-III-mediated abscission.^47^ This separates the cytoplasmic portion of the receptors from the rest of the cell, leading to abrogation of its signalling. Interestingly, depletion of ESCRT-0 and ESCRT-I subunits inhibits the degradation of EGFR and results in enhanced recycling and sustained activation of extracellular signal-regulated kinase (ERK) signalling.^48,49^

A role for endosomal acidification and ligand dissociation has also been established. Recycling of receptors to the plasma membrane takes place if the ligands are released in the early endosome (i.e., transferrin receptor), where the pH is maintained at ~6.5.^50^ Conversely, some signalling receptors (i.e., EGFR) often retain ligand binding and remain active even at low (~4.5) pH, leading to their continual signalling from endosomal compartments until they are sorted into ILVs and degraded in the lysosome.^51^

Some internalised receptors in early endosomes can be sorted to the TGN in a process called retrograde transport (i.e., mannose-6-phosphate receptors and several toxins such as Shiga, cholera and ricin). The TGN is a network of interconnected tubules and vesicles at the *trans*-face of the Golgi apparatus. It is essential for maintaining cellular homoeostasis and is known to play a crucial role in protein sorting or diverting proteins and lipids away from lysosomal degradation.

Mature late endosomes are approximately 250–1000 nm in diameter and are round/oval in shape. They are characterised by the presence of RAB7-GTPase, which is fundamental for the maturation of early-to-late endosomes and for the lysosomal biogenesis. Maturation of early-to-late endosomes depends on the formation of a hybrid RAB5/RAB7 endosome, wherein RAB7 is recruited to the early endosome by RAB5-GTP.^52^ Late endosomes undergo homotypic fusion reactions, grow in size and acquire more intraluminal vesicles. Once intralumenal vesicles containing late endosomes become enriched with RAB35, RAB27A, RAB27B and their effectors Slp4 and Slac2b, they fuse to plasma membrane to release exosomes.^37^ The released exosomes are small (40–100 nm in diameter), single membrane-bound vesicles that contain protein, DNA and RNA. Mostly, however, late endosomes move to the perinuclear area of the cell in the vicinity of lysosomes using dynein-dependent transport. Here, late endosomes undergo transient fusions with each other and eventually fuse with lysosomes to generate a transient hybrid organelle called the endolysosome. It is in the endolysosomes in which most of the hydrolysis of endocytosed cargo takes place.^37^ Following a further maturation process, the endolysosome is converted into a classical dense lysosome.

Cellular contents and organelles can also be delivered to lysosomes through a separate pathway called autophagy. Autophagy or self-eating is a unique membrane trafficking process whereby a newly formed isolation membrane can elongate and engulf part of the cytoplasm or organelles to form autophagosomes that are delivered to the lysosome for degradation. There are an increasing number of reports pointing to a mechanistic role for autophagy in the process of tumour metastasis, detailed in a recent review.^53^

An astonishing number of endosomal trafficking pathway proteins are known to be functionally important in tumour progression and metastasis (Table 1). Many have been validated in cancer cell motility and invasion, but a considerable number have been shown to modulate in vivo metastasis. The alterations identified include up- or down-regulation of expression, or mutation, and generally lead to an aberrant receptor trafficking/recycling/degradation/signal duration, which has a profound effect on cancer cell migration, invasion and/or proliferation. While most of these reports focus on a single signalling pathway, it is likely that multiple pathways are also affected. These mechanistic studies cover a wide range of cancer types. Additional details on different endosomal trafficking members and their role(s) in cancer and metastasis can be found in recent reviews.^54–56^

## Integrin and extracellular matrix trafficking in metastasis

Cancer cells invade through the extracellular matrix (ECM) in part by producing matrix metalloproteinases (MMPs) and other proteinases that degrade the ECM, thereby creating paths for migration. Similarly, cells attach to the ECM by means of integrins that are key regulators of cell adhesion, migration and proliferation. The interplay between integrins and ECM remodelling proteases is a major regulator of tumour invasion.

In oral squamous cell carcinoma (SCC), increased αVβ6 integrin expression leads to the activation of MMP-3 and promotes oral SCC cell proliferation and metastasis in vivo.^57^ MMP-14 (membrane type 1 metalloprotease MT1-MMP), along with integrin αVβ3 co-localised to the protruding ends of invadopodia, and its high local concentration on the cell membrane promoted metastasis.^58^ Interestingly, WDFY2 (a cytosolic protein) controls the recycling of MT1-MMP to the membrane, and loss of WDFY2 leads to enhanced secretion of MT1-MMP leading to active invasion of cells.^59^

Recent studies highlight the importance of integrin trafficking (endocytosis and recycling) as a modulator of cancer cells’ fate. For example, rapid recycling of integrins from the leading edge of individual cells assists in efficient cell motility by providing a supply of fresh receptors that are internalised at the trailing edge. More details on the trafficking of MMPs and integrins and its role in metastasis can be found in recent reviews.^60,61^

## Metastasis suppressors and endocytosis

Metastasis suppressors are a group of genes that suppress the metastatic potential of cancer cells without significantly affecting the size of primary tumour.^62^ So far, more than 20 metastasis suppressor genes (including miRNAs) have been identified in multiple cancer types with a wide range of biochemical activities.^63^ Some of the metastasis suppressor genes working through alterations in endocytosis are described below:

### NME1 (NM23/NM23-H1, non-metastatic clone 23, isoform H1)

NME is a multifunctional protein that is highly conserved from yeast to humans. Its enforced expression suppressed metastasis in a variety of cancer cell lines without altering primary tumour growth.^64^ Apart from being a metastasis suppressor, it is also known to have a developmental function.

The *Drosophila* homologue of NME is *awd* (*abnormal wing discs*) and is known to regulate cell differentiation and motility of multiple organs in late embryogenesis by regulating growth factor receptor signalling through endocytosis. These studies identified a genetic interaction between *awd* and *dynamin* (*shi*).^65^ An aberrant endocytosis was associated with mutant *awd* phenotypes and complemented RAB5 or *shi* genes.^65–67^ It was also shown that *awd* regulated tracheal cell motility in development by modulating the fibroblast growth factor receptor (FGFR) levels through *dynamin*-mediated endocytosis.^65,68^ Interestingly, loss of *awd* gene also blocked Notch signalling by altering the receptor processing that leads to Notch accumulation in the early endosomes.^67^

Recent reports in mammalian cancer models have also highlighted the role of NME as an interacting partner of Dynamin in endocytosis.^69,70^ NME transfectants of multiple cell lines exhibited increased endocytosis of EGFR and transferrin in concert with motility suppression. Both the increased endocytic and motility-suppression phenotypes were blocked by inhibitors of Dynamin. In a lung-metastasis assay, NME1 overexpression failed to significantly suppress metastasis in cells in which Dynamin 2 was also knocked down. Using the EGF/EGFR signalling axis as an in vitro model, NME1 decreased the phospho-EGFR and phospho-Akt levels in a Dynamin 2-dependent manner, highlighting the relevance of this interaction for downstream signalling. It was speculated that NME acted as a GTP provider/oligomerising agent of Dynamin 2, leading to higher Dynamin 2 GTPase activity and increased endocytosis (Fig. 1).^69,70^ Our data identified another function of a NME–Dynamin interaction: in vitro, NME promoted the oligomerisation of Dynamin and its increased GTPase activity, which are needed for vesicle scission.^69^

### KAI1 (CD82, cluster of differentiation 82)

KAI1/CD82 is a member of the evolutionarily conserved tetraspanin family, and was initially identified as a metastasis suppressor in prostate cancer.^71^ KAI1 has since been established as a metastasis suppressor in a variety of solid tumours. Its higher expression predicts a better prognosis,^72–74^ whereas reduced expression of KAI1 has been widely correlated with an aggressive cancer in several cancer types, including pancreatic, hepatocellular, bladder, breast and non-small-cell lung cancers.^73,75,76^

KAI1-mediated suppression of metastasis is thought to be achieved primarily by inhibiting cancer cell migration and invasion.^77^ This phenotype is the result of forming oligomeric complexes with binding partners such as integrins, EGFR and intracellular signalling proteins, such as protein kinase C (PKC). This complex generally leads to either redistribution or increased internalisation of multiple receptors. For example, overexpression of KAI1 leads to redistribution of urokinase-type plasminogen activator receptor (uPAR) into a stable complex with integrin α5β1 in focal adhesions.^78^ Focal adhesion binding of uPAR reduces its ability to bind the ligand uPA and consequently to cleave and activate plasminogen. Similarly, KAI1 also binds with EGFR, ErbB2 and ErbB3; for EGFR, this leads to accelerated endocytosis and desensitisation.^79,80^ KAI1 also specifically inhibits ligand-induced EGFR dimerisation and alters the distribution of EGFR in the plasma membrane, which consequently affects its activation.^80^

### MTSS1 (metastasis suppressor protein 1 or MIM, missing in metastasis)

MTSS1/MIM, originally identified in bladder cancer cell lines, was present in non-metastatic but not metastatic bladder cancer cells.^81^ It is hypothesised that MTSS1 suppresses metastasis by acting as a scaffold protein to interact with actin-associated proteins to regulate cytoskeletal dynamics and lamellipodia formation, consequently affecting invasion and metastatic behaviour of cancer cells.^82^ In head and neck squamous cell carcinoma, MTSS1 augments EGF signalling by antagonising EGFR endocytosis at low cell densities and promotes cellular proliferation at early stages of primary head and neck squamous cell carcinoma tumour growth. However, at high cell densities, MTSS1 has a negative impact on EGF signalling and inhibits metastasis.^83^

### KISS1 (kisspeptin-1)

The KISS1 gene produces a peptide product called kisspeptins (KP), which act as an endogenous ligand for a G-protein-coupled receptor, KISS1R (GPR54).^84^ KISS1 acts as a metastasis suppressor gene through its KP/KISS1R signalling in numerous human cancers (melanoma, pancreatic cancer and gastric carcinoma) by inhibiting cellular motility, proliferation, invasion, chemotaxis and metastasis.^85^ However, in breast cancer, KP stimulates invasion of cancer cells and high expression of KISS1; GPR54 mRNA levels positively correlated with shorter relapse-free survival. Interestingly, GPR54 directly complexes with EGFR, and stimulation of breast cancer cells by either EGF or KP-10 regulated the endocytosis of both GPR54 and EGFR.^86^ This signalling has an opposite effect on breast cancer cells, i.e., it is pro-migratory and pro-invasive in human breast cancer cells.

Metastasis suppressor genes, while often showing statistically significant inverse trends of tumour expression and patient survival, are not likely to become clinically used prognostic factors, in favour of more complex gene signatures. As with tumour suppressors, their translation to the clinic has also been problematic. Restoration of metastasis suppressor expression in every metastatic tumour cell would be needed for optimal activity, which is unrealistic. Our laboratory explored the transcriptional upregulation of NME by high-dose medroxyprogesterone acetate.^87^ A Phase 2 trial, conducted at Indiana University, was a technical failure, as serum levels of medroxyprogesterone acetate were not sufficiently elevated, although some long-term stable disease was observed.^88^ How the endocytic pathways can contribute to a metastatic-suppressor clinical–translational effort is currently unknown but of high interest. More research to identify the complex mechanisms underlying these processes is warranted.

## Conclusions

Endocytosis is a process of internalisation of the plasma membrane along with its membrane proteins and lipids. Cells use endocytosis to regulate signalling and to sample the extracellular milieu for appropriate responses. It affects almost all of the steps of metastasis and is used as a tool for the functioning of metastasis suppressors. Based on the literature, endocytosis regulates receptor internalisation, recycling and degradation, or could affect cytoskeleton dynamics to alter cancer cell invasion or metastasis. However, the majority of the above conclusions have been made based on studies conducted on cancer cell lines. These studies would benefit from validation on patient-derived tissues. Other challenges in this field are a lack of high-resolution knowledge of the endosomal sorting complexes and their central regulators, and how signalling in cancer cells is altered at specific stages of endocytosis. These issues will undoubtedly be clarified as research progresses. Identification of these central regulators could serve as trafficking nodes that are amenable to therapeutic interception. A potential issue in translation is the effect of an inhibitor of an endocytic node on multiple signalling pathways that it engages, and how the cumulative effects modulate the metastatic phenotype. This issue is not unique to endocytosis and applies to DNA methylation and other cancer processes. In summary, targeting the endocytic machinery could be a viable and promising therapeutic strategy for cancer and metastasis.

## Data Availability

Not applicable.
